# Anti-GD2 mAb and Vorinostat synergize in the treatment of neuroblastoma

**DOI:** 10.1080/2162402X.2016.1164919

**Published:** 2016-03-28

**Authors:** Michiel Kroesen, Christian Büll, Paul R. Gielen, Ingrid C. Brok, Inna Armandari, Melissa Wassink, Maaike W. G. Looman, Louis Boon, Martijn H. den Brok, Peter M. Hoogerbrugge, Gosse J. Adema

**Affiliations:** a Department of Tumor Immunology, Radboud Institute for Molecular Life Sciences, Radboud University Medical Center, Nijmegen, The Netherlands; b Department of Pediatric Oncology, Radboud University Medical Center, Nijmegen, The Netherlands; c EPIRUS Biopharmaceuticals Netherlands B.V., Utrecht, The Netherlands; d Princes Máxima Center for Pediatric Oncology, De Bilt, The Netherlands

**Keywords:** Anti-gd2 mAb therapy, histone deacytelase inhibitor, immunotherapy, myeloid-derived suppressor cell, neuroblastoma

## Abstract

Neuroblastoma (NBL) is a childhood malignancy of the sympathetic nervous system. For high-risk NBL patients, the mortality rate is still over 50%, despite intensive multimodal treatment. Anti-GD2 monoclonal antibody (mAB) in combination with systemic cytokine immunotherapy has shown clinical efficacy in high-risk NBL patients. Targeted therapy using histone deacetylase inhibitors (HDACi) is currently being explored in cancer treatment and already shows promising results. Using our recently developed transplantable TH-MYCN NBL model, we here report that the HDAC inhibitor Vorinostat synergizes with anti-GD2 mAb therapy in reducing NBL tumor growth. Further mechanistic studies uncovered multiple mechanisms for the observed synergy, including Vorinostat-induced specific NBL cell death and upregulation of the tumor antigen GD2 on the cell surface of surviving NBL cells. Moreover, Vorinostat created a permissive tumor microenvironment (TME) for tumor-directed mAb therapy by increasing macrophage effector cells expressing high levels of Fc-receptors (FcR) and decreasing the number and function of myeloid-derived suppressor cells (MDSC). Collectively, these data imply further testing of other epigenetic modulators with immunotherapy and provide a strong basis for clinical testing of anti-GD2 plus Vorinostat combination therapy in NBL patients.

## Abbreviations

ADCC: antibody dependent cellular cytotoxicity
ADCP: antibody dependent cellular phagocytosis
APC: antigen presenting cell
BMDM: bone marrow derived macrophage
DC: dendritic cells
FcR: Fc-receptor
GD2: disialoganglioside
HDACi: histone deacetylase inhibitor
mAb: monoclonal antibody
MDSC: myeloid-derived suppressor cell
MHCI: MHC Class I
MHCII: MHC Class II
M-MDSC: monocytic myeloid-derived suppressor cell
NBL: neuroblastoma
NK cells: natural killer cells
PMN-MDSC: polymorphonuclear myeloid-derived suppressor cell
TIL: tumor infiltrating leukocytes
TME: tumor microenvironment
Treg: T regulatory cell.

## Introduction

NBL is a childhood malignancy of the sympathetic nervous system accounting for 12% of cancer-associated deaths in children under 15 y of age.^1^ Patients with high-risk NBL, including *MYCN*-amplified NBL, have a poor long-term survival despite intensive multimodal treatment.^2^ In a recent phase III clinical trial, immunotherapy improved the event-free survival of high-risk NBL patients by around 20%.^3^ The immunotherapy in this trial consisted of a tumor-specific mAb directed toward the tumor antigen GD2 in combination with administration of immune stimulating cytokines IL-2 and GM-CSF. FcR expressing immune cells, including natural killer (NK) cells, granulocytes and recently also other myeloid cells, have been implicated as the effector cells in the clinical response following anti-GD2 mAb.^4,5^ However, despite the observed clinical benefit using immunotherapy, still about half of the patients show progressive disease. Therefore, further improvement of NBL treatment is needed to increase the survival of these pediatric patients.

One approach to improve NBL treatment efficacy is to combine anti-GD2 mAb immunotherapy with tumor-targeted therapy. The ultimate goal of such an immunocombination therapy is to induce and boost potent antitumor immunity and to counteract tumor-induced immune suppression, as reviewed in ref.^6^. An emerging tumor-targeting therapy involves the use of HDACi as epigenetic modulators to eliminate and modulate tumor cells.^7^ HDAC inhibition results in increased acetylation of histone proteins leading to changes in gene expression.^8^ HDACi target multiple cellular processes simultaneously and also impact normal cells, although tumor cells appear much more sensitive.^9^ Specifically in cancer cells, the altered gene expression leads to activation of and sensitization to intrinsic and extrinsic apoptosis pathways.^10^ Besides targeting histone proteins, HDACi also induce hyperacetylation of pro-apoptotic cytosolic proteins like p53, thereby also inducing tumor cell death.^11^ The classical HDACi block the function of one or more of the 11 classical zinc-containing HDAC enzymes. Among the zinc-containing HDACs are the class-I HDAC (HDAC 1,2,3, and 8) and class-II HDAC (HDAC 4,5,6,7,9,10) enzymes.^12^ Class specific HDACi inhibit HDAC from either class, while panHDACi inhibit HDACs from both classes. Various panHDACi are currently in phase I–III trials for the treatment of cancer.^13,14^

To design novel strategies that combine immunotherapy with tumor targeted therapies, we generated a transplantable autologous NBL model derived from the TH-MYCN transgenic mouse. The *MYCN* proto-oncogene is frequently amplified on the genomic level in NBL, a phenomenon associated with an adverse prognosis.^15,16^ The TH-MYCN transgenic mouse model is driven by over expression of N-MYC in developing sympathetic nervous cells and closely resembles high-risk human NBL.^17,18^ Using our transplantable TH-MYCN model in C57Bl/6 mice, we found that the immunobiology of this model was highly similar to human NBL, including endogenous expression of the tumor surface antigen GD2.^19^ Moreover, similar to NBL in patients, the NBL tumors arising in the TH-MYCN NBL model were highly infiltrated by myeloid cells, including macrophages and MDSC, suggestive for an important role in NBL pathogenesis.^19-21^ Macrophages in tumors are generally classified as either antitumor M1 or pro-tumor M2 macrophages.^22,23^ MDSC are immature myeloid cells that accumulate in tumors and can mediate potent local and systemic immune suppression.^24^

In the current study, we report that anti-GD2 mAb therapy combined with the HDACi Vorinostat results in synergistic antitumor effects in this novel NBL mouse model. As part of the explanation of this synergy, we uncovered that TH-MYCN NBL cells were highly sensitive to HDACi-mediated cell death, while surviving NBL cells upregulated the tumor antigen GD2. Furthermore, Vorinostat treatment altered the composition and function of myeloid cells in NBL tumors, resulting in myeloid cells expressing less immune suppressive genes and more activating FcR. Our study provides a rationale for clinical testing of GD2 mAb plus Vorinostat combination therapy in NBL patients.

## Results

### TH-MYCN NBL cells are highly sensitive to HDACi-mediated cell death

**Figure 1.**
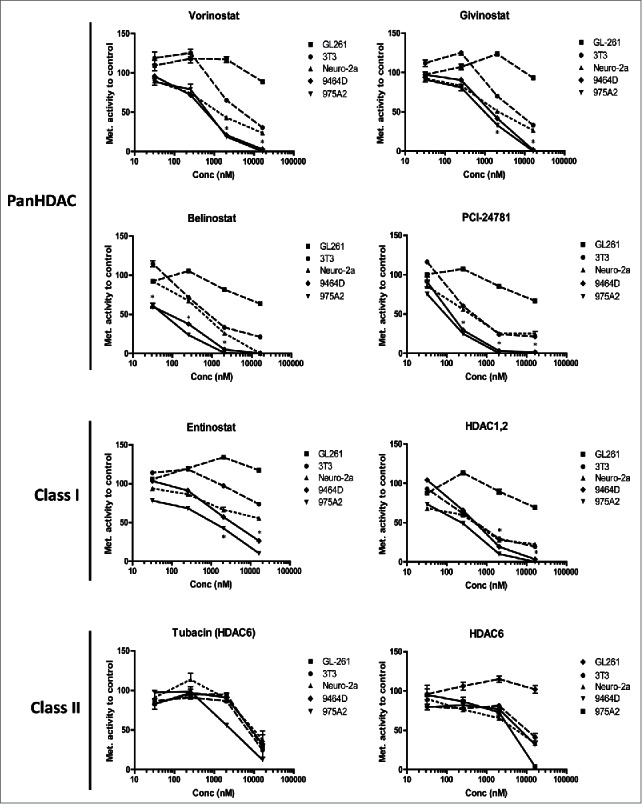
Neuroblastoma cells are sensitive to HDACi-mediated cell death. (A) TH-MYCN derived 9464D and 975A2 neuroblastoma cells, Neuro-2a neuroblastoma, GL261 glioblastoma and 3T3 fibrosarcoma cells were incubated for 36 h with 32, 256, 2048 and 16384 nM of the indicated HDACi. Following a 36 h incubation, standard MTT assays were performed, metabolic activity was compared to control treated cells and plotted in dose response curves (*p < 0.05 for 9464D or 975A2 vs. Neuro-2a or GL261 or 3T3). Representative graphs of three independent experiments are shown.

To determine whether the murine TH-MYCN cell lines 9464D and 975A2 were sensitive to HDACi-mediated cell death, these cells were exposed to increasing concentrations of various HDACi, after which viability was determined via standard MTT metabolic activity assays. For comparison, the *MYCN*-negative NBL Neuro-2a, the glioblastoma GL261 and the fibrosarcoma 3T3 cell lines were used. TH-MYCN *Mycn*-transgenic 9464D and 975A2 NBL cells, incubated with the panHDACi Vorinostat, Givinostat, Belinostat and PCI-24781, showed a significantly stronger reduction in metabolic activity and hence in viability compared to the non-*MYCN* NBL cell line Neuro-2a and the other non-NBL cell lines GL261 and 3T3 (Fig. 1). Subsequent analysis revealed that the 9464D and 975A2 NBL cells were also more sensitive for the class-I specific HDACi Entinostat and a HDAC1,2 specific HDACi compared to the control cell lines (Fig. 1). In contrast, the class-II HDACi Tubacin and a HDAC6 specific HDACi had little impact on either the TH-MYCN cells or the control tumor cell lines (Fig. 1). The half maximal inhibitory concentrations (IC50) for the different HDACi and cell lines tested are depicted in Table 1. These IC50 values and 95% confidence intervals demonstrate that the murine TH-MYCN NBL cells are highly sensitive to pan- and class-I HDACi when directly compared to other non-NBL murine cancer cell lines and the non-*MYCN* NBL cell line Neuro-2a.

**Table 1.** IC50s (in nM) for the various HDACi and cell lines are depicted with corresponding 95% confidence intervals.

	GL261	3T3	Neuro-2a	9464D	975A2
Vorinostat	>16,000	3,535 (2,175–5,745)	1,446 (882–2,372)	608 (540–683)	657 (446–969)
Givinostat	>16,000	5,111 (3,122–8,368)	2,426 (1,603–3,671)	1504 (1,207–1,874)	980 (753–1,275)
Belinostat	>16,000	627 (396–992)	579 (482–695)	84 (54–130)	58 (47–71)
PCI-24781	>16,000	363.7 (225–587)	406 (208–794)	138 (96–199)	89 (79–102)
Entinostat	>16,000	>16,000	13,453 (6,941–26,075)	2,997 (2,245–4,000)	1,001 (555–1,804)
HDAC1,2	>16,000	587 (360–957)	408 (184–903)	468.5 (393–558)	194.8 (141–297)
Tubacin	9,362 (5,416–16,085)	7,207 (4,710–11,029)	4,064 (6,010–22,358)	9,710 (4,222–22,382)	2,737 (2,198–3,408)
HDAC6	>16,000	9,948 (4,925–20,093)	4,807 (2,182–10,591)	10,837 (3,584–12,652)	4,099 (1,830–6,459)

### Anti-GD2 mAb plus vorinostat combination therapy is synergistic in reducing NBL growth

**Figure 2.**
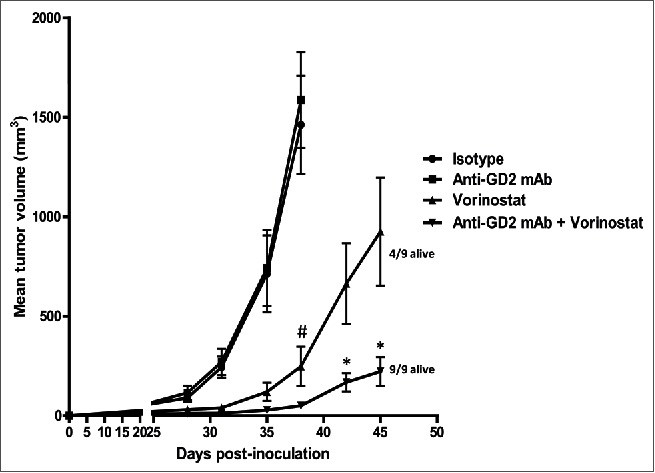
Anti-GD2 mAb and Vorinostat mediate synergistic anti-NBL effects *in vivo*. Immunocombination therapy using anti-GD2 mAb and Vorinostat results in synergistic inhibition of NBL tumor growth. (A) Mice were inoculated s.c. with 1 × 10^6^ 9464D cells on day 0. Anti-GD2 mAb therapy (200 μg/mouse, i.p.) was initiated on day 8 and repeated twice weekly. Vorinostat treatment (150 mg/kg, i.p.) was initiated on day 14 and given for 3 consecutive days and this scheme was repeated weekly until day 45. Tumor growth was monitored and tumor volumes were calculated. Occasionally (< 5% of all mice), tumors caused skin ulceration which was randomly divided over the treatment groups; these mice were sacrificed and excluded from the analysis. Mean tumor volumes for each treatment group (9 mice/group) are depicted (^#^*p* < 0.05 for isotype or anti-GD2 vs. Vorinostat or Vorinostat + anti-GD2) (*p < 0.05 for Vorinostat vs. Vorinostat + anti-GD2). On day 45, 9/9 mice of the anti-GD2 plus Vorinostat group, whereas 4/9 mice of the Vorinostat monotherapy group were still alive (defined by tumor volume < 1000 mm^3^). Representative data of two independent are shown.

Next, we determined the effect of anti-GD2 mAb treatment alone or in combination with HDACi treatment at stringent therapeutic conditions *in vivo*. We selected the panHDACi Vorinostat for these studies as it strongly reduced the viability of 9464D NBL cells and has entered phase I clinical trials in pediatric oncology patients, including NBL patients (www.clinicaltrials.gov).^14^ Mice bearing established 9464D NBL tumors were treated with anti-GD2 mAb or Vorinostat alone and with the combination of both. Anti-GD2 mAb monotherapy was initiated on day 8 following tumor inoculation and was repeated twice weekly until day 43. In this stringent therapeutic model, anti-GD2 mAb treatment alone, starting on day 8 post-inoculation, had no or very little impact on tumor growth relative to isotype control Ab (Fig. 2). As we recently reviewed, pre-activated effector immune cells are further enhanced in their function by HDAC inhibitors, providing a rationale to initiate HDAC inhibitor therapy after immunotherapy.^25^ Therefore, we started Vorinostat treatment after anti-GD2 therapy in our experiments. I.p. injections of Vorinostat (150 mg/kg) were administered daily for 3 consecutive days and this treatment schedule was repeated weekly, until day 45. Vorinostat monotherapy caused a significant reduction in tumor growth compared to PBS/DMSO control treatment (Fig. 2). Interestingly, the combination of Vorinostat plus anti-GD2 mAb therapy resulted in a synergistic reduction of tumor growth in this therapeutic setting (Fig. 2). On day 45, the last day of the combination treatment, all (9/9) mice in the anti-GD2 plus Vorinostat combination group versus 4/9 mice in the Vorinostat monotherapy group were still alive. We concluded that the combination of anti-GD2 mAb-based immunotherapy and targeted therapy using Vorinostat-reduced NBL tumor growth in a synergistic manner.

### Vorinostat increases GD2 expression on NBL cells and anti-GD2 mAb mediated killing

**Figure 3.**
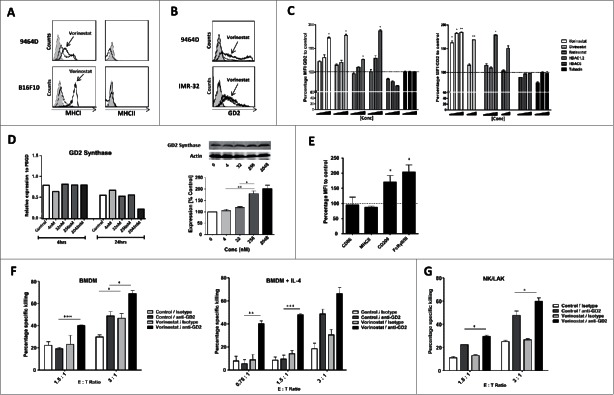
HDACi increase GD2 expression by NBL cells resulting in increased anti-GD2 mAb-mediated killing. (A) Expression of MHCI and MHCII by 9464D and B16F10 cells following incubation with 256 nM Vorinostat or control for 18 h. Gray shading = isotype control, Thin line = control treated, Thick line = Vorinostat treated. Representative data from three independent experiments are shown. (B) Expression of GD2 by 9464D and IMR-32 cells after incubation with 2.5 μM and 256 nM Vorinostat, respectively, for 18 h. Gray shading = isotype control, Thick line = specific staining. Representative data from three independent experiments are shown. (C) Expression of GD2 relative to control by 9464D and IMR-32 cells after incubation with the indicated HDACi for 18 h. Mean Fluorescence Intensity (MFI) of GD2 is depicted. Pooled data from three independent experiments are depicted (*p < 0.05, **p < 0.01). (D) 9464D cells were exposed to indicated concentrations of Vorinostat for 24 h after which cells were lysed and analyzed by qPCR (left) and Western Blot (right) for expression of GD2 Synthase (*p < 0.05, **p < 0.01). Representative data from two independent experiments are depicted. (E) Day 6 BMDM were treated with 20 ng/mL IL-4 for 24 h and analyzed for the expression of CD86, MHCII, CD206 and FcRγ2/3. Pooled data from two independent experiments are depicted (*p < 0.05). (F,G) Treatment of 9464D cells with Vorinostat increases anti-GD2 mAb mediated killing. 9464D cells were incubated for 18 h with 256 nM Vorinostat or control and then co-cultured with indicated effector cells in the presence of isotype control Ab or anti-GD2 mAb (*p < 0.05, **p < 0.01, ***p < 0.001). Representative data from 2–3 independent experiments for each immune effector cell type are shown.

To uncover the mechanisms responsible for the observed synergy of anti-GD2 mAb plus Vorinostat combination therapy *in vivo*, we first investigated the effect of Vorinostat on the expression of immune relevant antigens on surface of the tumor cells *in vitro*. Hereto, tumor cells were exposed for 18 h to Vorinostat in concentrations which affected viability only mildly, maximally 20%. The data revealed that Vorinostat treatment upregulated MHC class I (MHCI), but not MHC class II (MHCII) expression, on surviving 9464D NBL and B16F10 melanoma cells (Fig. 3A). The expression of other immune-related molecules tested, including the NK cell activating ligands Rae1 and Mult-1 as well as the co-inhibitory molecule PD-L1, was not altered following HDACi exposure (data not shown).

Next, we determined whether the expression of the GD2 antigen itself was affected by Vorinostat treatment. As previously reported, 9464D NBL cells shows a population with an upregulated expression of GD2 and a population with a very high expression of GD2 (Fig. 3B).^26^ Strikingly, Vorinostat increased GD2 expression in both populations of the murine 9464D as well as in human IMR-32 NBL cells, resulting in a more than 150% increase in the Mean Fluorescent Intensity of GD2 compared to control treated cells (Figs. 3B and C). We also observed a dose dependent upregulation of GD2 by the panHDACi Givinostat, the class-I inhibitor Entinostat and a HDAC1,2 specific inhibitor (Fig. 3C). In contrast, GD2 levels were not increased by the class-II HDACi Tubacin or a HDAC6 specific inhibitor (Fig. 3C). Next, the effect of increasing concentrations of Vorinostat on the transcription of *GD2 Synthase*, an essential gene for GD2 expression, was assessed using qPCR.^27,28^ No significant changes were observed in *GD2 Synthase* mRNA levels in 9464D cells following Vorinostat treatment (Fig. 3D, left). Vorinostat exposure, however, did result in increased GD2 Synthase protein levels in a dose-dependent manner (Fig. 3D, right).

To determine the functional consequences of increased GD2 expression, 9464D cells were treated with Vorinostat or control for 24 h, washed and subsequently co-cultured with immune effector cells in the presence of anti-GD2 mAb or an isotype control Ab. As effector cells, BM derived macrophages (BMDM) treated with or without IL-4 and NK/LAK cells were used. Addition of IL-4 upregulated CD206 and FcRy1/3 expression in BMDM, suggestive of an M2-like phenotype with enhanced phagocytic capacity (Fig. 3E).^29,30^ Vorinostat pre-treated tumor cells were not killed more efficiently by BMDM or NK cells relative to control treated tumor cells (Figs. 3F,G). Addition of anti-GD2 mAb, but not isotype control mAb, however, resulted in significantly enhanced killing of the Vorinostat pre-treated tumor cells relative to control cells irrespective of the type of immune effector cell (Figs. 3F,G). Interestingly, IL-4 treated, M2-like BMDM mediated anti-GD2 mAb mediated killing of Vorinostat pre-treated tumor cells even at low effector : target ratio's (Fig. 3F, right). Collectively, these data indicate that Vorinostat treatment of NBL cells enhanced GD2 expression on NBL cells, resulting in more efficient anti-GD2 mAb-mediated tumor cell killing by NK cells, macrophages and especially M2-like macrophages.

### Vorinostat increases GD2 expression and affects the composition of tumor myeloid cells

**Figure 4.**
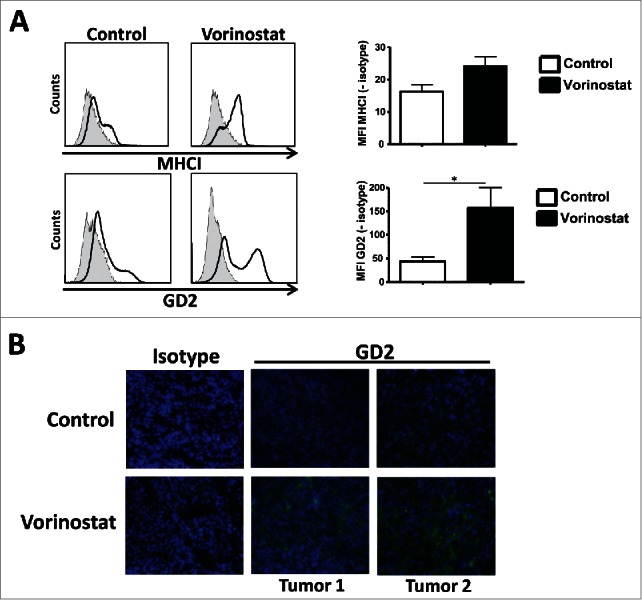
Vorinostat increases GD2 expression by NBL cells *in vivo*. (A) Mice bearing 9464D tumors (6 mice/group) were treated with Vorinostat (150 mg/kg) for 3 consecutive days. One day after the last injection tumors were excised and single-cell suspensions were made. Expression of MHCI and GD2 was determined on the CD45.2^−^ tumor cells for Vorinostat and control tumors (*p < 0.05). Representative data from three independent experiments. (B) Cryo-sections of 9464D tumors treated with Vorinostat or control were stained using isotype control Ab or anti-GD2-Cy5 mAb. Representative data from two independent experiments are presented.

To determine whether Vorinostat could also increase GD2 expression *in vivo*, mice-bearing NBL tumors were treated with Vorinostat or DMSO/PBS control. Similar to our *in vitro* observations, GD2 expression on tumors was significantly increased following Vorinostat treatment as measured by FACS (Fig. 4A) and immunohistochemistry (Fig. 4B). Vorinostat treatment also seemed to upregulate the expression of MHCI on the tumor cells *in vivo*, but this did not reach statistical significance (Fig. 4A).

**Figure 5.**
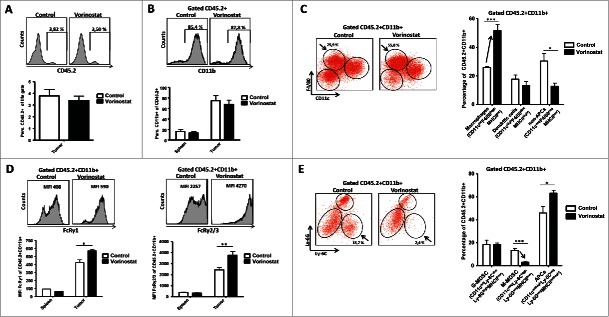
Vorinostat treatment increases the presence of macrophages while reducing M-MDSC in the TME of NBL tumors. Mice bearing 9464D tumors (6 mice/group) were treated with Vorinostat (150 mg/kg) for 3 consecutive days after which tumors were excised and single-cell suspensions were made. (A) Vorinostat does not alter total leukocyte infiltration of NBL tumors. The total tumor cell suspension was analyzed for the presence of CD45.2^+^ leukocytes. Representative data from three independent experiments. (B) Vorinostat does not alter myeloid cell presence in spleens and tumors. CD45.2^+^ leukocytes were gated and analyzed for the expression of CD11b. Representative data from three independent experiments are shown. (C) Vorinostat increases the presence of macrophages within the tumor infiltrating myeloid cells. CD45.2^+^CD11b^+^ tumor-infiltrating myeloid cells were gated and analyzed for the expression of CD11c, F4/80 and MHCII. Percentages of CD11c^dim^F4/80^high^MHCII^int^ macrophages, CD11c^high^F4/80^dim^MHCII^high^ DC and CD11c^low^F4/80^low^MHCII^low^ non-APC are depicted (*p < 0.05, ***p < 0.001). Representative data from three independent experiments are presented. (D) Vorinostat upregulates the expression of FcRγ1 and FcRγ2/3 on the cell surface of tumor-infiltrating myeloid cells. CD45.2^+^CD11b^+^ myeloid cells were gated and analyzed for the expression of FcRγ1 and FcRγ2/3 (*p< 0.05, **p< 0.01). Representative data from three independent experiments. (E) Vorinostat reduces M-MDSC within the tumor-infiltrating myeloid cells. CD45.2^+^CD11b^+^ myeloid cells were gated and analyzed for the expression of CD11c, Ly-6C, Ly-6G and MHCII. Percentages of and CD11c^neg^Ly-6C^high^Ly-6G^neg^MHCII^low^ M-MDSC, CD11c^low/int^Ly-6C^dim^Ly-6G^high^MHCII^low^ PMN-MDSC and CD11c^int/high^Ly-6C^neg^Ly-6G^neg^MHCII^int/high^ APCs are depicted (*p < 0.05; ***p < 0.001). Representative data from three independent experiments are shown.

Effects of Vorinostat on tumor cells have been extensively studied, but much less are known regarding its effect on immune cell infiltration and function in tumors *in vivo*. Therefore, we determined the presence and phenotype of immune cells in the NBL TME following Vorinostat treatment. Percentages of CD45.2^+^ tumor infiltrating leukocytes (TIL) within the total tumor cell suspension were unaltered in Vorinostat and control treated tumors (Fig. 5A). Within these CD45.2^+^ TIL, the percentages of CD3^+^CD4^+^ and CD3^+^CD8^+^ T cells, CD3^−^NK1.1^+^ NK cells and CD11b^+^ myeloid cells were all very similar in Vorinostat and control treated tumors (Fig. S1A and Fig. 5B). T cell subset analysis revealed that also the level of CD4^+^FoxP3^+^ Treg within the CD4^+^ T cell population was not affected by Vorinostat treatment (Fig. S1B).

Analysis of the tumor infiltrating CD45.2^+^CD11b^+^ myeloid cells showed a significant increase in the percentage of CD11c^dim^F4/80^high^MHCII^int^ macrophages upon Vorinostat treatment (Fig. 5C). In contrast, there was a strong decrease in the CD11c^low^F4/80^low^MHCII^low^ non-APC, while the percentage of CD11c^high^F4/80^dim^MHCII^high^ DC was not altered (Fig. 5C). These data indicate the presence of more macrophages in NBL tumors following Vorinostat treatment. Strikingly, these macrophages expressed high levels of FcRy1 and FcRy2/3 resulting in a significantly increased expression of these FcR in the total CD45^+^CD11b^+^ myeloid population (Fig. S2 and Fig. 5D). The CD11c^dim^F4/80^high^MHCII^int^ macrophages after Vorinostat treatment showed increased surface expression of CD206 and CD80, while expression of MHCII was significantly decreased (Fig. S3). These data suggest a mixed M1/M2 phenotype of these tumor macrophages following Vorinostat treatment.

We next analyzed the tumor-infiltrating myeloid cells using mAbs directed toward the markers Ly-6C and Ly-6G to discriminate between polymorphonuclear MDSC (PMN-MDSC) and monocytic MDSC (M-MDSC), respectively.^31^ Strikingly, CD11c^neg^Ly-6C^high^Ly-6G^neg^MHCII^low^ cells with a phenotype corresponding to M-MDSC were largely depleted from Vorinostat treated tumors (Fig. 5E). The percentage of CD11c^int/high^Ly-6C^neg^Ly-6G^neg^MHCII^int/high^ cells with a phenotype corresponding to APC was increased, while the percentage of CD11c^low/int^Ly-6C^dim^Ly-6G^high^MHCII^low^ cells corresponding to PMN-MDSC was unaltered (Fig. 5E). Thus, Vorinostat treatment significantly changes the composition of the tumor-infiltrating myeloid cells in these NBL tumors, resulting in more macrophages showing a mixed M1/M2 phenotype and less M-MDSC.

### Vorinostat creates a more permissive TME for tumor-directed mAb therapy

To determine the functional consequences of Vorinostat treatment on the tumor-infiltrating myeloid cells in NBL tumors, myeloid cells were isolated from tumors of mice treated with Vorinostat or control. First, CD45.2^+^ TIL from pooled tumor cell suspensions of 18 mice per treatment arm were isolated using MACS. Next, the CD45.2^+^CD11b^+^ myeloid cells were purified to homogeneity by FACsort and directly lysed for RNA extraction. RT-qPCR analysis of the house keeping genes *Gusb* and *Pbgd* relative to each other showed unaltered expression levels for control and Vorinostat treated samples, indicating these housekeeping genes could be used for relative gene expression analysis following Vorinostat treatment (Fig. S4).

**Figure 6.**
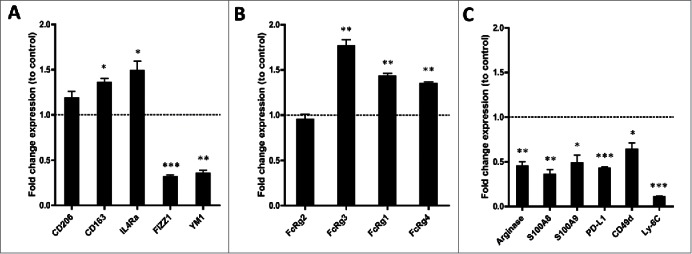
Vorinostat treatment results in a TME-containing myeloid cells expressing more activating FcR and less immune suppressive genes. (A–D) Mice (18 mice/group) bearing 9464D tumors were treated with Vorinostat (150 mg/kg) for 3 consecutive days. One day after the last injection tumors were excised and single-cell suspensions were made and pooled. CD45.2^+^ TILs were isolated from the pooled tumor cell suspensions by CD45.2^+^ MACS separation. The CD45.2^+^CD11b^+^ cells were subsequently FACS-sorted and directly lysed for RNA isolation. Following cDNA synthesis, qPCR analysis was performed in triplicate and mRNA expression levels relative to Pbgd were determined for the indicated genes. mRNA expression relative to Pbgd of the treatment groups were normalized to control samples and are presented as fold-change relative to control (*p < 0.05, **p < 0.01, ***p < 0.001).

To better understand the nature of the Vorinostat-induced myeloid cells in NBL tumors, the expression of commonly used macrophage markers was determined. The transcript levels of the M2 macrophage markers *Cd206, Cd163, Il4ra* were all increased, whereas the levels of M2 markers *Fizz1* and *Ym1*, were decreased in the tumor-infiltrating myeloid cells following Vorinostat, suggesting a mixed M1/M2 macrophage phenotype (Fig. 6A). In addition, we determined the expression of several cytokines by the tumor myeloid cells following Vorinostat treatment. Expression of the M2-associated cytokine TGF-β was not altered following Vorinostat treatment, whereas the M2-associated cytokine IL-10 was significantly increased (Fig. S5). The M1-associated cytokines TNF-α and IL-6 were both significantly decreased (Fig. S5). These data are in line with the aforementioned FACS data comparing control versus Vorinostat-induced macrophages (Fig. S3).

Next, we determined the expression of genes related to myeloid cell function, like FcR essential for ADCC and immunosuppressive mediators. The FACS analysis already revealed a significant increase in the levels of FcRy1 and FcRy2/3 expressed on CD45.2^+^CD11b^+^ myeloid cells in the tumor (Fig. 5D). The latter mAb, however, recognizes both FcRy2b and FcRy3, and does not discriminate between inhibiting and activating FcR.^32-34^ Vorinostat treatment induced an increase in mRNA levels of the activating, low-affinity *Fcrg3*, while expression of the inhibitory, low-affinity *Fcrg2b* was not altered (Fig. 6B). Moreover, the high-affinity, transcripts of the activating receptors *Fcrg1* and *Fcrg4* were both increased in the tumor infiltrating myeloid cells upon Vorinostat treatment (Fig. 6B). In addition, transcript levels of four genes directly involved in immune suppression, *Arginase, S100a8*, *S100a9 and Pdl1*, were all strongly decreased in the myeloid cells of Vorinostat treated tumors (Fig. 6C).^31^ In agreement with the reduced expression of these immunosuppressive genes, the markers *Cd49d* and *Ly6c*, expressed mainly by immunosuppressive M-MDSC, were also expressed to a much lesser extent in the myeloid compartment of Vorinostat treated tumors (Fig. 6D).^35,36^ Collectively, these data indicate that Vorinostat treatment creates an immune permissive TME for tumor-directed mAb therapy in NBL tumors.

## Discussion

Here, we report that anti-GD2 mAb plus Vorinostat combination therapy mediates synergistic antitumor effects in a novel autologous NBL mouse model. As an explanation for this synergy, we uncovered that TH-MYCN transgenic NBL cells were highly sensitive to HDACi-mediated cell death. In addition, the HDACi Vorinostat upregulated the GD2 antigen on surviving NBL cells resulting in enhanced anti-GD2 mAb mediated killing. Finally, Vorinostat treatment completely altered the NBL TME, resulting in more macrophages expressing activating FcR and less M-MDSC expressing immune suppressive genes. These mechanistic insights into anti-GD2 plus Vorinostat combination therapy provide a rationale for clinical testing of this combination therapy in NBL patients.

Over the past decade, several tumor-targeted mAbs were shown to induce clinical responses.^6,37^ Durable clinical responses following tumor-specific mAb therapy, however, are observed in only 10–30% of cancer patients.^38^ Previously, we observed an initial delay in the outgrowth of NBL tumor upon GD2 mAb therapy in our transplantable model, but in the current more stringent setting no significant delay in overall survival was observed.^19^ Similar to these observations in mice, anti-GD2 mAb therapy alone did not have a major clinical effect in NBL patients when combined with Retinoic Acid therapy, a vitamin A metabolite inducing NBL differentiation.^39^ Anti-GD2 mAb plus systemic cytokines IL-2 and GM-CSF and retinoic acid therapy, however, resulted in a 20% improved 5 y survival.^3^ We now show that the efficacy of anti-GD2 mAb therapy for NBL in mice is also enhanced by addition of the HDACi Vorinostat. Interestingly, the combination of the panHDACi Valproic acid and Retinoic Acid was previously shown to induce synergistic NBL differentiation and apoptosis.^40^ Therefore, it will be extremely interesting to determine how addition of epigenetic modulators will affect the efficacy of anti-GD2 mAb therapy plus Retinoic Acid/IL-2/GM-CSF combination therapy. Vorinostat combined with Retinoic Acid caused grade 3 hematologic toxicity at maximum in the six evaluated patients in a phase I trial, whereas systemic IL-2 and GM-CSF administration caused grade 3 to 4 capillary leak syndrome and hypotension in around 20% and 10% of patients. Because of these apparent different toxicity profiles, it may also be important to explore Vorinostat plus anti-GD2 mAb combination therapy in the absence of systemic cytokine administration.^3,14^

As an explanation for the synergy of Vorinostat plus anti-GD2 mAb combination therapy, we found novel effects of Vorinostat treatment on NBL cells and immune cells *in vivo. MYCN* transgenic NBL cells were highly sensitive for panHDACi and Class-I HDACi. One possible explanation for this high sensitivity are the reported interactions of N-MYC with several class-I HDACs.^41,42^ In addition to inducing efficient NBL cell death, Vorinostat treatment increased the expression of the tumor antigen GD2 on surviving NBL cells, resulting in enhanced anti-GD2 mAb-mediated tumor cell killing *in vitro*. How Vorinostat treatment affects GD2 expression levels is not yet clear. The finding that Vorinostat did not induce significant changes in *GD2 Synthase* mRNA levels in 9464D cells, but did increase GD2 Synthase protein levels, implies that Vorinostat either acts via affecting the direct acetylation of GD2 Synthase or indirectly via (transcriptional) regulation of *GD2 Synthase* (de)stabilizing genes.

Immune suppressive Treg and MDSC can hamper the function of immune effector cells in tumors.^43^ Several panHDACi were previously shown to increase the number and function of immune suppressive Treg.^44,45^ Following our Vorinostat treatment regimen, we did not observe increased Treg numbers. Vorinostat rather seemed to decrease FoxP3 expression levels in Treg, a phenomenon previously reported for the class-I HDACi Entinostat (Fig. S1C).^46^ MDSC are immature myeloid cells that can actively inhibit antitumor immune responses and accumulate in NBL tumors in TH-MYCN transgenic mice.^20,21,24,36,47^ Our experiments now show that Vorinostat treatment essentially eliminates the presence of M-MDSC from these NBL tumors. Moreover, Vorinostat treatment reduced levels of *Arginase* and many other genes implicated in M-MDSC-mediated immune suppression within the CD45^+^CD11b^+^ NBL tumor-infiltrating myeloid cells. These data are therefore indicative for diminished tumor immune suppression as a consequence of Vorinostat treatment.^36,48^

FcR expressing immune effector cells are highly important for the clinical response following tumor-directed mAb therapy, including anti-GD2 mAb therapy.^4,5,49,50^ We did not observe differences in the number of NK cells infiltrating NBL tumors after Vorinostat treatment (Fig. S1A). We, however, did observe increased numbers of macrophages in NBL tumors, expressing high levels of the activating FcRy1 and FcRy2/3 on their cell surface following Vorinostat treatment. Expression of the activating receptors *Fcrg1, Fcrg4* and *Fcrg3*, but not the inhibitory receptor *Fcrg2b*, was also increased at the mRNA level.

Analysis of the macrophage type induced by Vorinostat treatment revealed a mixed M1/M2 macrophage gene signature as well as surface and cytokine expression profile. Although M2 type macrophages in NBL tumors were previously associated with an adverse disease outcome, tumor-infiltrating macrophages as a whole were repeatedly shown to be important immune effector cells, especially following tumor-directed mAb therapy.^51-54^ In addition, recent expression profiling studies indicate that the M1 and M2 division for macrophages is a serious oversimplification of the complexity of macrophage subtypes and functions in tumors.^22,23^ Our data and other studies support that at least part of the M2 macrophage characteristics are not by default negative for cancer therapy, and may even be necessary for effective antitumor mAb therapy.^30^

Overall, our data imply that enhanced expression of GD2, lower numbers of immune suppressive M-MDSC and higher numbers of FcR^high^-expressing macrophages in the tumor all contribute to the efficacy of the Vorinostat plus anti-GD2 mAb combination therapy. Further support for these immune effects of Vorinostat is provided by our preliminary data showing enhanced capacity of total CD45.2^+^ TIL present in Vorinostat treated tumors to eliminate 9464D tumor cells relative to the total TIL isolated from control tumors.

The synergy of anti-GD2 mAb therapy with Vorinostat reported here, may have important implications for NBL patients. As both anti-GD2 mAb plus cytokine treatment and Vorinostat therapy are applied in pediatric oncology patients, our study provides a rationale for immunocombination treatment with Vorinostat and anti-GD2 mAb therapy in the treatment of NBL patients.^14^

A few studies have now reported on successful combinations of HDACi with immunotherapy.^46,55,56^ One aspect that deserves further study is the optimal timing of HDACi treatment in immunocombination therapy, as this may critically determine the efficacy *in vivo*.^46^ In our experiments, anti-GD2 mAb was administered prior to Vorinostat. Whether this order of administration is crucial for the observed synergy in anti-GD2 mAb plus Vorinostat combination therapy and whether the same order is required for T-cell-based immunotherapies remains to investigated.

## Materials and methods

### Animals and cell lines

Six–eight weeks old female C57Bl/6 wild-type (WT) mice held under specified pathogen-free conditions in the Central Animal Laboratory (Nijmegen, the Netherlands) were purchased from Charles River (Sulzfeld, Germany). All experiments were performed according to the guidelines for animal care of the Nijmegen Animal Experiments Committee. GL261 cells were provided by U. Herrlinger (Bonn, Germany) in 2006 and were not re-authenticated by the author. IMR-32, Neuro-2a, 3T3 and B16F10 cell lines were obtained from and authenticated by ATCC (CCL-127, CCL-131, CRL-1658, CRL-6475, respectively) (Manassas, VA). 9464D and 975A2 cells were derived from spontaneous tumors from TH-MYCN transgenic mice on C57Bl/6 background and were a kind gift from Dr Orentas in 2010 (NIH, Bethesda) and were authenticated by the author last in 2013 by qPCR using primers against NBL specific genes, including *Mycn* and *GD2 synthase*. All cell lines were initially grown and multiple aliquots were cryopreserved and used within 6 mo after resuscitation and tested for mycoplasma using a mycoplasma detection kit (Lonza, Basel, Switzerland). The hybridoma 14.g2a producing a mouse IgG2a mAb specific for GD2 was a kind gift from Dr Reisfeld (Scripps, La Jolla). B16F10 cells were cultured in Minimal Essential Medium (MEM) containing 5% fetal bovine serum (FBS, Greiner Bio-one, Alphen a/d Rijn, the Netherlands), 1% non-essential amino acids (NEAA) (Invitrogen), 0.5% antibiotic-antimycotic (AA) (Gibco), 1% pyruvate (Gibco), 2% NaH_2_CO_3_ (Gibco), 1.5% MEM Vitamins (Gibco) and 0.05% β-mercaptoethanol. GL261 cells were cultured in Iscove's Modified Dulbecco's Medium (IMDM) (Gibco) containing 10% FCS, 1% NEAA, 0.5% AA and 0.05% β-mercaptoethanol. Neuro-2a, IMR-32, 9464D, 975A2 and 3T3 cell lines were cultured in Dulbecco's modified Eagle (DMEM) medium containing 10% FCS, 1% NEAA, 0.5% AA and 0.05% β-mercaptoethanol.

### HDACi

HDACi were purchased from SelleckChem, Houston, USA. An HDAC1,2 specific HDACi was synthesized similar to compound 5a in ref.^57^. The HDAC6-specific HDACi was synthesized similar to compound 7 in ref.^58^. All HDACi were dissolved in DMSO to a final concentration of 5 mM. For *in vivo* studies Vorinostat was dissolved in DMSO/PBS 50 mg/mL.

### Tumor induction

For induction of tumors, 1 × 10^6^ 9464D cells were injected s.c. in 50 μL PBS on the right flank of mice. Tumor cell viability before injection was >95%. As determined by Trypan Blue staining. Tumor growth was measured every 3–4 d using calipers. Tumor volume was calculated with the formula: (A × B^2^) × 0.4 in which A is the largest and B is the shortest dimension.

### Anti-GD2 mAb and vorinostat treatment in vivo

Anti-GD2/control treatment started on day 8 following tumor inoculation by i.p. injection of 200 μg mAb and was repeated twice weekly until day 43. Vorinostat treatment started on day 14 following tumor inoculation by injecting 150 mg/kg Vorinostat or DMSO/PBS control i.p. for 3 consecutive days and was repeated every week until day 45.

### Vorinostat treatment and single-cell suspensions

Mice bearing 9464D tumors of 300–600 mm^3^ were injected i.p. with 150 mg/kg Vorinostat or DMSO/PBS for 3 consecutive days. Tumors excised one day following the last injection, were mechanically dissociated, and enzymatically digested with 1 mg/mL collagenase Type III (Worthington) and 30 μg/mL DNAse type I (Roche) for 1 h at 37°C. EDTA was added to 12.5 mM and fragments were passaged over 100 μm cell strainers. For GD2 staining *ex vivo*, cell suspensions were washed with PBS to decrease background staining.

### Antibodies and flow cytometry

Anti-GD2 (clone 14.g2a) mAb was purified and filter sterilized in PBS. Total mouse IgG control Ab was obtained from Jackson Immunoresearch. Directly labeled mAbs used for staining were CD11b-A700 (M1/70), H-2K^b^/H-2D^b^-PE (28-8-2006), F4/80-PE-Cy7 (BM8), CD11c-PerCP (N418), CD4-PerCP (L3T4), Ly-6G-PE-Cy7 (1A8), CD64-PE (X54-5/7.1), CD16/CD32-A647 (2.4G2), CD206-APC (MR5D3) from Biolegend, CD45.2-FITC (104), CD11c-APC (HL3), NK1.1-PE (PK136), Ly-6C-PE (AL-21) and CD3-APC (145-2C11) from BD, MHC II-PE (M5/114.15.2) and FoxP3-PE-Cy7 (FJK-16s) from eBioscience, CD8-A700 (53–6.7) from Exbio. Cells for staining were washed in PBS, incubated with Viability Dye eFluor 780 (eBioscience), resuspended in PBA, transferred to a V-bottom 96-wells plate and stained using specific mAb or the appropriate isotypes. Cells analyzed on a Cyan apparatus (Beckman Coulter) using Summit software.

### CD45.2^+^ MACS and FACSort

Mice (18 mice/group) bearing 9464D tumors were treated i.p. with 150 mg/kg Vorinostat or DMSO/PBS for 3 consecutive days. Tumors excised one day following the last injection and pooled single-cell suspensions were resuspended in MACS buffer (1.5 mM EDTA, 1% FBS). Cells were passaged over a washed MACS Column (Miltenyi) to remove aggregates, followed by a CD45.2 MACS according to the manufacturer's protocol. Purified cells were stained with anti-CD11b-A700 (M1/70) and CD45.2^+^CD11b^+^ cells were subsequently FACsorted to homogeneity on a FACS Aria (BD) with a typical purity of >98%.

### Quantitative PCR

CD45.2^+^CD11b^+^ FACS-sorted cells were lysed and RNA was isolated and quantified by spectrophotometry. cDNA was synthesized using random primers and Moloney murine leukemia virus Superscript Reverse Transcriptase (II-MMLV) (Invitrogen). Relative mRNA levels were determined with a Biorad CFX96 cycler using the Fast Start SYBR Green Kit (Invitrogen). Intron spanning primers (Sigma-Aldrich) were designed and tested in our lab and are provided upon request Data were analyzed with Bio-rad CFX manager version 1.6 (Bio-rad) and checked for correct amplification and dissociation of the PCR-products. PBGD and GUSB served as reference genes. mRNA expression was determined relative to PBGD expression using the formula 2^(CT index – CT PBGD)^.

### MTT assay

Cells were seeded in flat-bottom 96-well plates (Costar) at 5 × 10^3^ cells/well and cultured overnight to adhere. HDACi were added to final concentrations of 32, 256, 2048, and 16348 nM. After 36 h incubation, medium was refreshed and 10 µL MTT reagent (4 mg/mL) (Sigma) was added in PBS. Plates were incubated for 2–4 h at 37°C after which supernatant was removed and 100 µL lysis buffer (0.5% SDS, 4% HCl, and 3.5% Milli-Q in isopropanol) added. After 30 min of incubation, absorbance was measured using an ELISA reader (Bio-Rad) at 595 nm. Metabolic activity versus control was calculated as (treatment – blank)/(control – blank) × 100%. Triplicate wells for each concentration were performed.

### Generation of NK/LAK effector cells

NK/LAK cells were generated from splenocytes of naive mice. A 10-mL syringe was filled with 1.2 g of Nylon Wool (Polysciences) and sterilized. Five spleens were collected from 6–8 weeks old naive female C57Bl/6 WT mice and single-cell suspensions were made by passage over a 100 μm cell strainer. Ery-lysis was performed using ACK-buffer and stopped with complete medium. The nylon wool “column” was prepared using IMDM, containing 5% FCS, 1% AA, 2 mM Glutamine and 0.05% β-mercaptoethanol (medium) for 1 h at RT. Splenocytes were passaged over a nylon wool column and were plated at a density of 2.5 × 10^6^ cells/mL in medium containing 1000 IU/mL of human rIL-2. On day 3 and 6, non-adherent cells were washed away by extensive medium washings. On day 7, adherent cells were harvested using 1.5 mM EDTA. The harvested adherent cells were >90% NK1.1^+^NKp46^+^ cells, as determined by flow cytometry.

### Generation of bone marrow derived macrophages (BMDM)

Bone marrow cells were isolated from the femurs of 6–8 weeks old female C57Bl/6 WT mice. 4 × 10^6^ bone marrow cells were cultured in a 10 cm^2^ petridish (Gibco) in medium supplemented with 20 ng/mL M-CSF (Peprotech). On day 3 and day 6, fresh M-CSF was added to the culture. When indicated, 20 ng/mL IL-4 (Peprotech) was added on day 6 to the BMDM culture to obtain M2 BMDM. On day 7, the non-adherent cells were discarded and washed away and adherent cells were harvested using cold 1.5 mM EDTA for experimental use.

### FACS-based killing assay

9464D cells were incubated for 24 h in the presence of 256 nM Vorinostat or control. 9464D (target) cells were harvested and labeled with 1 μM CFSE (Invitrogen) according to the manufacturer's protocol. Labeled target cells were plated at 20.000 cells/well in a flat bottom 96-well plate. Target cells were pre-incubated with medium containing 10 μg/mL anti-GD2 mAb or isotype control for 1 h at 4°C. Effector cells NK/LAK, BMDM or BMDM treated with IL-4 for 24 h, were added in effector: target cell ratios of 2:1, 4:1 and 6:1 for NK/LAK and 0,75:1, 1,5:1 and 3:1 for BMDM. After 8 h of co-culture, cells were harvested and stained with 7-AAD (BD) and Annexin-V-Cy5 (BD) according to the manufacturer's protocol. Triplicate samples were analyzed using a Cyan flow cytometer and the percentages of 7-AAD^+^, Annexin-V^+^ and 7-AAD^+^Annexin-V^+^ of CFSE^+^ target cells were determined. Percentage specific killing was calculated using the formula: (percentage of total dead target cells^target^ – percentage of total dead target cells^control^)/(100 – percentage of total dead target cells^control^) × 100.

### Immunohistofluorescence

Mice bearing established 9464D tumors were treated Vorinostat or DMSO/PBS control after which tumors were excised and snap frozen. Cryo-sections were made and directly fixated in −20°C acetone. Sections were stained with anti-GD2-Cy5 mAb or isotype control mIgG2a-Cy5 (HOPC-1) for 1 h at RT. Nuclear staining was performed using DAPI (Sigma) Sections were imaged using a Leica DM fluorescence microscope and analyzed using ImageJ software.

### Western blot analysis

9464D cells were incubated for 24 h with 0–2048nM Vorinostat and lysed in lysis buffer (150 mM NaCl, 5 mM EDTA, 50 mM TRIS pH 7.5 and 1% Triton X-100, 1 × protease inhibitor cocktail). For western blot analysis, samples were separated on 8% SDS-PAGE resolving gels and transferred to PVDF membranes. Membranes were incubated overnight at 4°C with primary anti-GD2-Synthase (Novus Biologicals, Littleton, CO) or anti-actin (Sigma-Aldrich, St. Louis, MO) antibodies in blocking buffer (1 × PBS, 0.25% bovine serum albumin, 0.5% non-fat dry milk). After washing with PBS-T (1 × PBS, 0.1% Tween-20), they were incubated with secondary IRDye 680LT-conjugated polyclonal goat anti-rabbit IgG (H&L) antibody (LI-COR, Lincoln, NE) in blocking buffer 1 h at RT. Membranes were washed thoroughly with PBS-T, scanned with an Odyssey infrared imaging system (LI-COR, Lincoln, NE) and band intensities were quantified using ImageJ (NIH, Bethesda, MD).

### Statistics

Data were analyzed using Graphpad 5.0 software. An unpaired T-test was used to determine significant differences between two groups. ANOVA test with a Bonferroni post-test was used to determine significant differences between three or more groups. IC50s were calculated by fitting log (inhibitor) – normalized response curves.

## Supplementary Material

KONI_A_1164919_s02.zip
