# Supplementary material for: Anti-GD2 mAb and Vorinostat synergize in the treatment of neuroblastoma
Source: Oncoimmunology. 2016 Mar 28;5(6):e1164919. doi: 10.1080/2162402X.2016.1164919 (PMC4938306; doi:10.1080/2162402X.2016.1164919)
Supplement: KONI_A_1164919_s02.zip [file koni-05-06-1164919-s001.zip › 2015ONCOIMM0693R-f11-z-bw.pptx]

## Slide 1
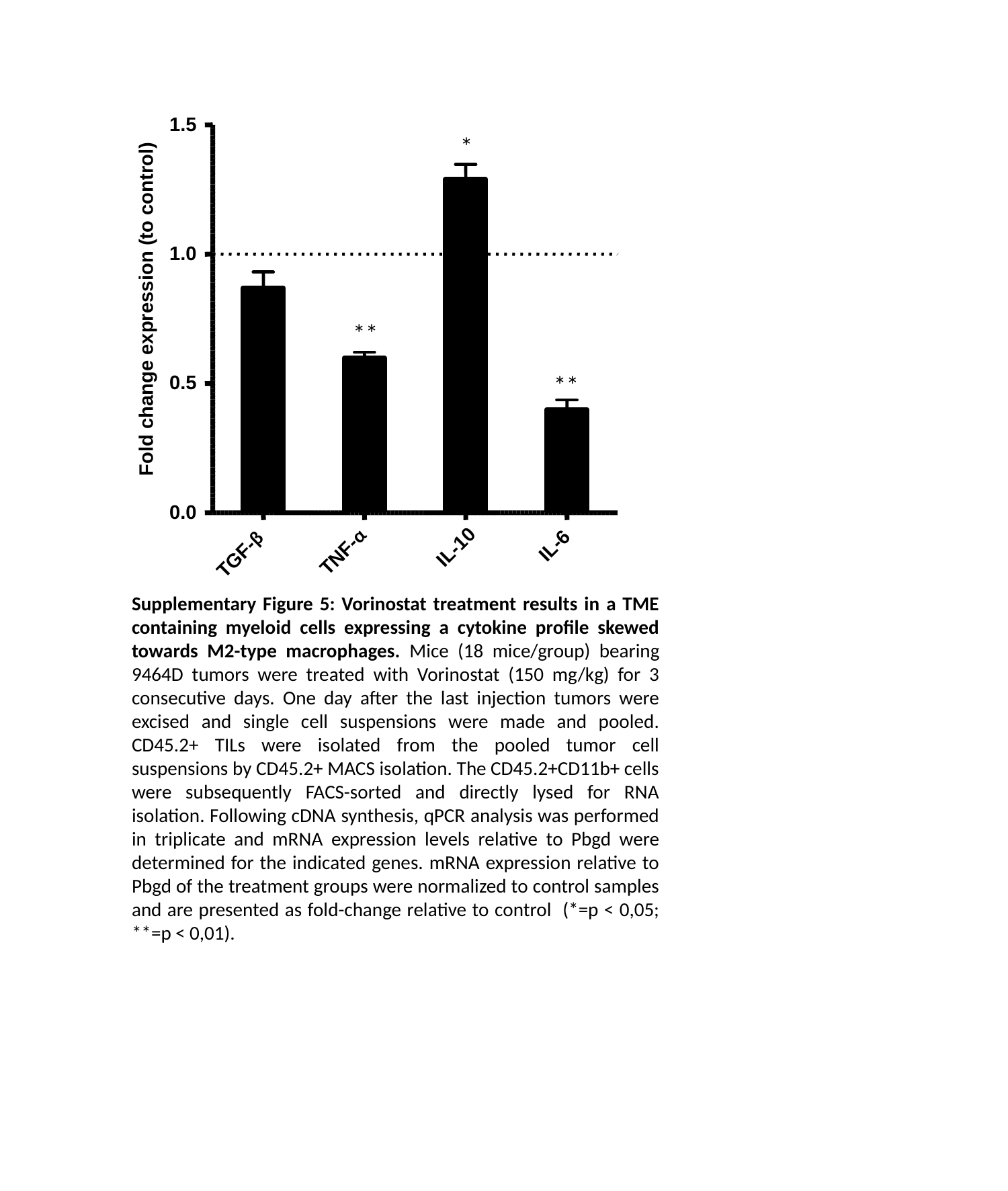

1.5
IL-6
IL-10
TNF-α
TGF-β
1.0
Fold change expression (to control)
0.5
0.0
*
**
**
Supplementary Figure 5: Vorinostat treatment results in a TME containing myeloid cells expressing a cytokine profile skewed towards M2-type macrophages. Mice (18 mice/group) bearing 9464D tumors were treated with Vorinostat (150 mg/kg) for 3 consecutive days. One day after the last injection tumors were excised and single cell suspensions were made and pooled. CD45.2+ TILs were isolated from the pooled tumor cell suspensions by CD45.2+ MACS isolation. The CD45.2+CD11b+ cells were subsequently FACS-sorted and directly lysed for RNA isolation. Following cDNA synthesis, qPCR analysis was performed in triplicate and mRNA expression levels relative to Pbgd were determined for the indicated genes. mRNA expression relative to Pbgd of the treatment groups were normalized to control samples and are presented as fold-change relative to control (*=p < 0,05; **=p < 0,01).
