# Supplementary material for: Anti-GD2 mAb and Vorinostat synergize in the treatment of neuroblastoma
Source: Oncoimmunology. 2016 Mar 28;5(6):e1164919. doi: 10.1080/2162402X.2016.1164919 (PMC4938306; doi:10.1080/2162402X.2016.1164919)
Supplement: KONI_A_1164919_s02.zip [file koni-05-06-1164919-s001.zip › 2015ONCOIMM0693R-f10-z-bw.pptx]

## Slide 1
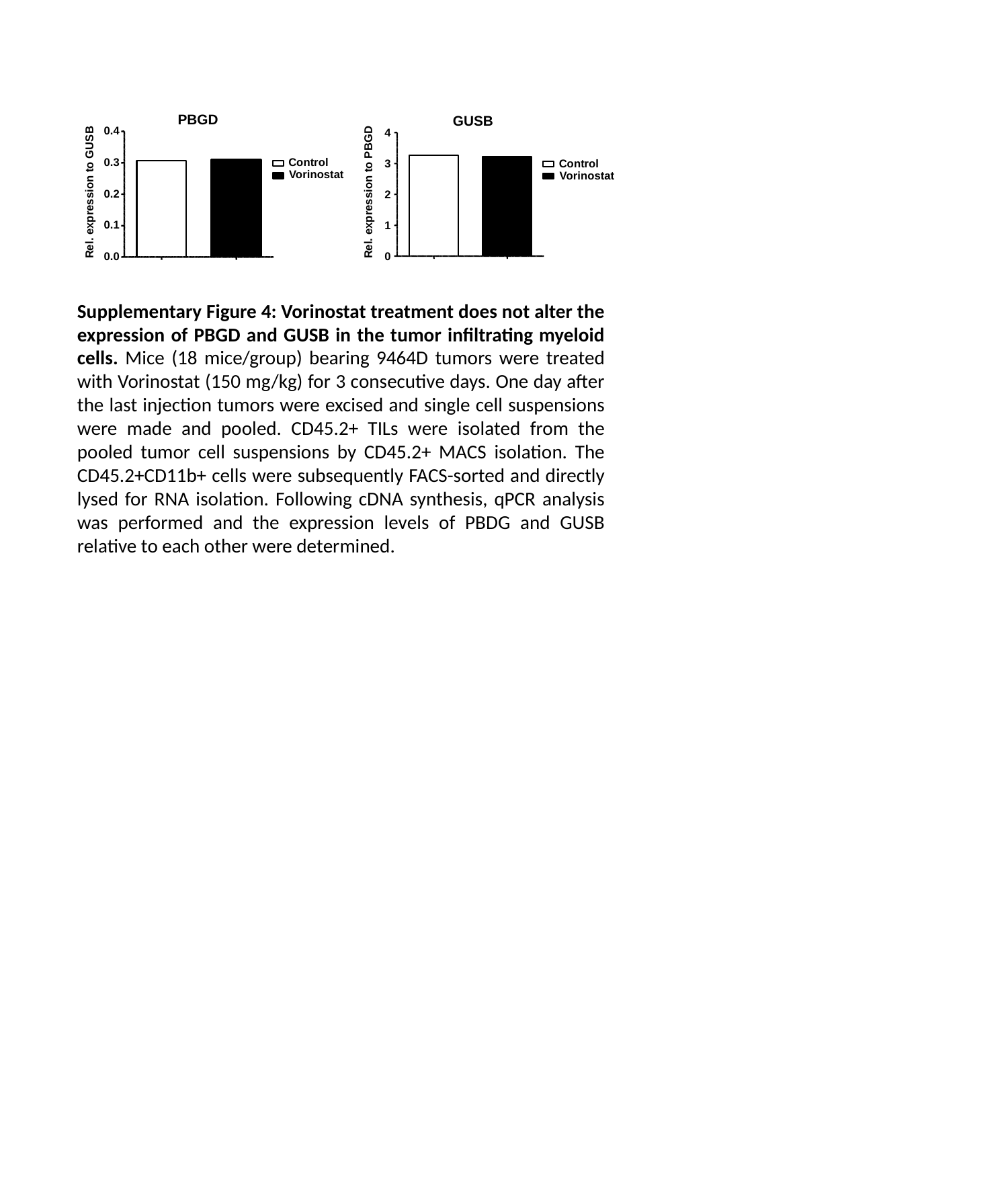

PBGD
0.2
0.1
0.0
0.4
0.3
Control
Vorinostat
Rel. expression to GUSB
GUSB
2
1
0
4
3
Control
Vorinostat
Rel. expression to PBGD
Supplementary Figure 4: Vorinostat treatment does not alter the expression of PBGD and GUSB in the tumor infiltrating myeloid cells. Mice (18 mice/group) bearing 9464D tumors were treated with Vorinostat (150 mg/kg) for 3 consecutive days. One day after the last injection tumors were excised and single cell suspensions were made and pooled. CD45.2+ TILs were isolated from the pooled tumor cell suspensions by CD45.2+ MACS isolation. The CD45.2+CD11b+ cells were subsequently FACS-sorted and directly lysed for RNA isolation. Following cDNA synthesis, qPCR analysis was performed and the expression levels of PBDG and GUSB relative to each other were determined.
