# Supplementary material for: Anti-GD2 mAb and Vorinostat synergize in the treatment of neuroblastoma
Source: Oncoimmunology. 2016 Mar 28;5(6):e1164919. doi: 10.1080/2162402X.2016.1164919 (PMC4938306; doi:10.1080/2162402X.2016.1164919)
Supplement: KONI_A_1164919_s02.zip [file koni-05-06-1164919-s001.zip › 2015ONCOIMM0693R-f09-z-bw.pptx]

## Slide 1
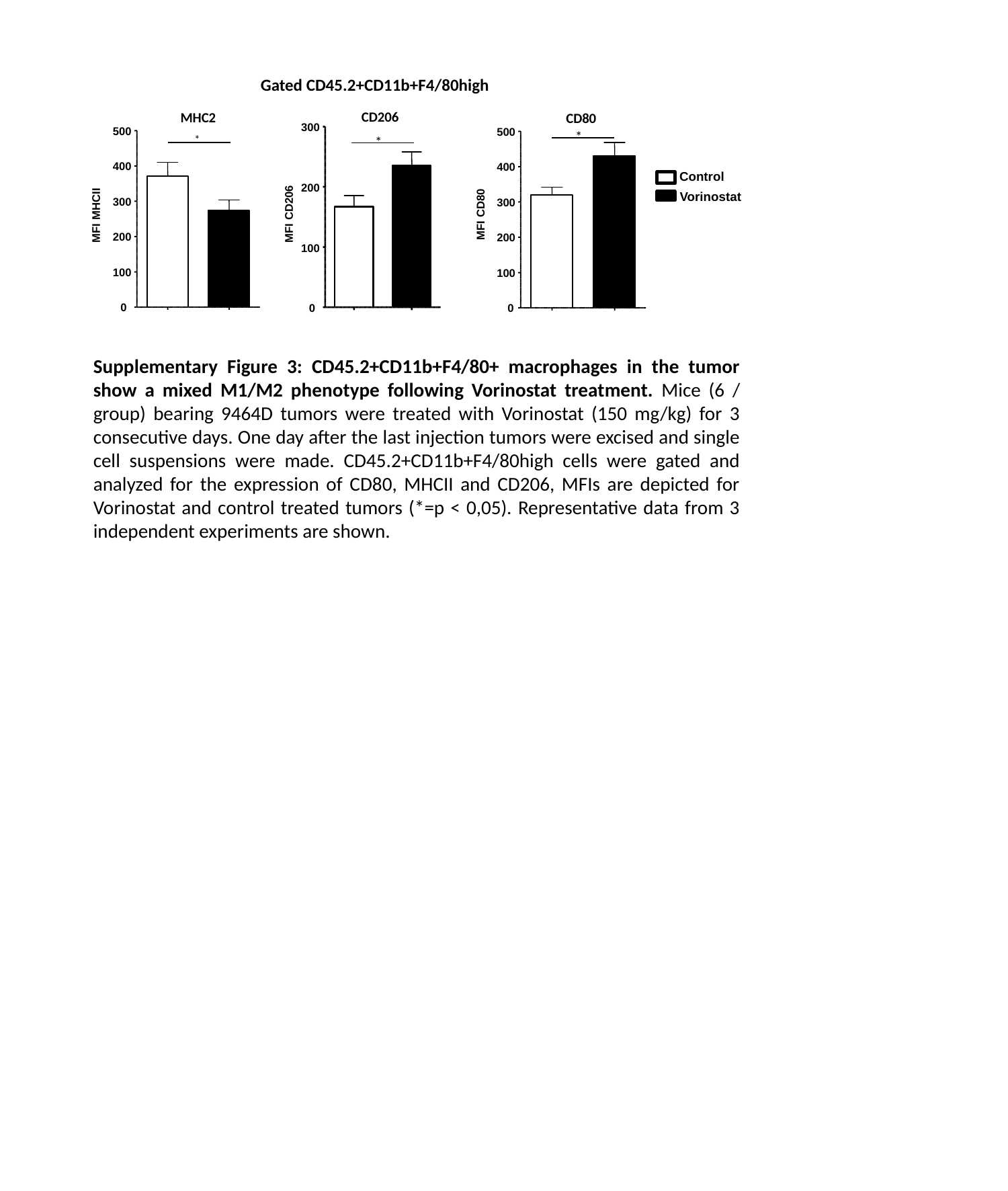

Gated CD45.2+CD11b+F4/80high
CD206
300
*
200
MFI CD206
100
0
MHC2
500
400
300
MFI MHCII
200
100
0
*
CD80
*
500
400
300
MFI CD80
200
100
0
Control
Vorinostat
Supplementary Figure 3: CD45.2+CD11b+F4/80+ macrophages in the tumor show a mixed M1/M2 phenotype following Vorinostat treatment. Mice (6 / group) bearing 9464D tumors were treated with Vorinostat (150 mg/kg) for 3 consecutive days. One day after the last injection tumors were excised and single cell suspensions were made. CD45.2+CD11b+F4/80high cells were gated and analyzed for the expression of CD80, MHCII and CD206, MFIs are depicted for Vorinostat and control treated tumors (*=p < 0,05). Representative data from 3 independent experiments are shown.
