# Supplementary material for: Anti-GD2 mAb and Vorinostat synergize in the treatment of neuroblastoma
Source: Oncoimmunology. 2016 Mar 28;5(6):e1164919. doi: 10.1080/2162402X.2016.1164919 (PMC4938306; doi:10.1080/2162402X.2016.1164919)
Supplement: KONI_A_1164919_s02.zip [file koni-05-06-1164919-s001.zip › 2015ONCOIMM0693R-f08-z-bw.pptx]

## Slide 1
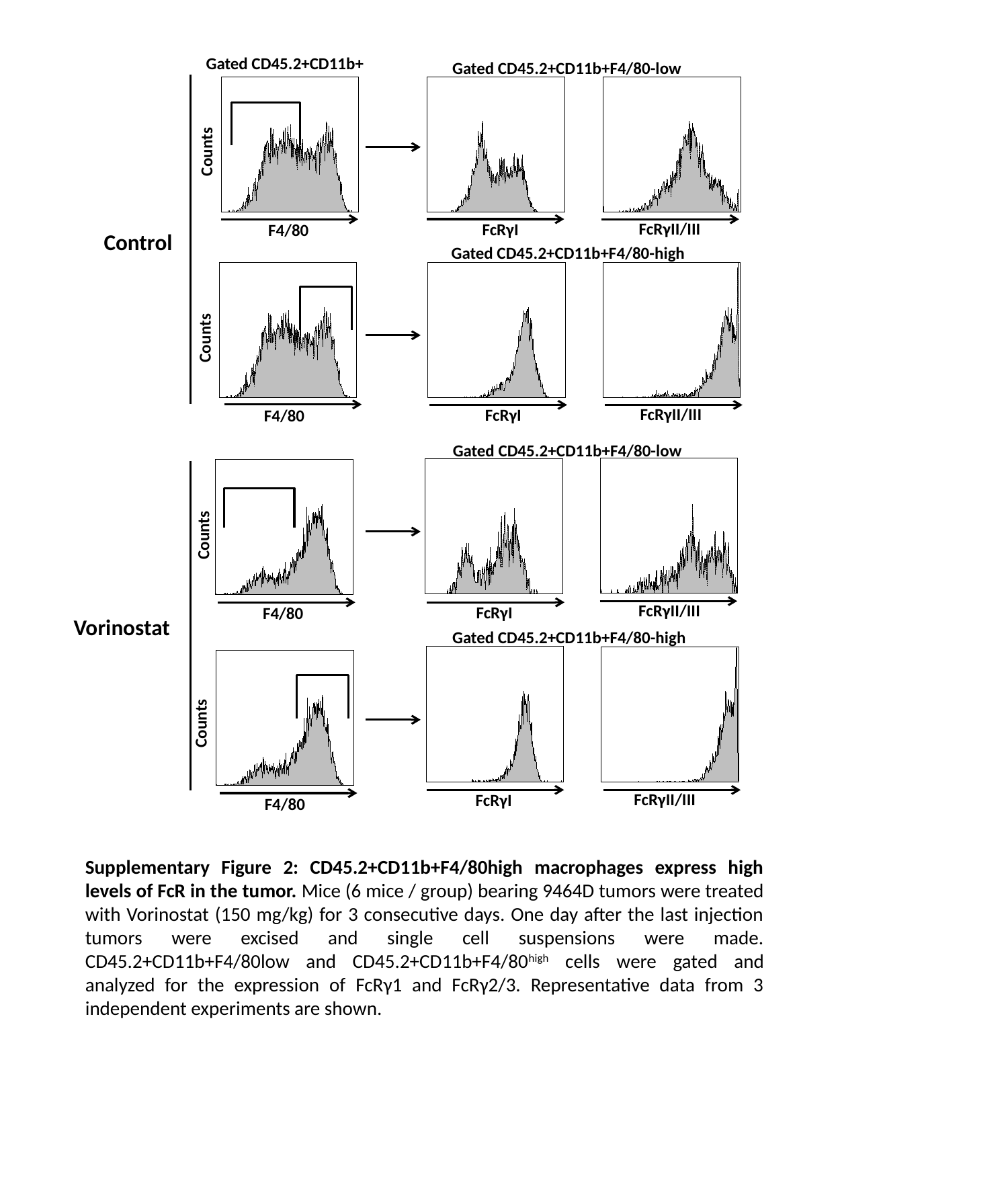

Gated CD45.2+CD11b+
Gated CD45.2+CD11b+F4/80-low
Counts
FcRγII/III
FcRγI
F4/80
Control
Gated CD45.2+CD11b+F4/80-high
Counts
FcRγII/III
FcRγI
F4/80
Gated CD45.2+CD11b+F4/80-low
Counts
FcRγII/III
FcRγI
F4/80
Vorinostat
Gated CD45.2+CD11b+F4/80-high
Counts
FcRγII/III
FcRγI
F4/80
Supplementary Figure 2: CD45.2+CD11b+F4/80high macrophages express high levels of FcR in the tumor. Mice (6 mice / group) bearing 9464D tumors were treated with Vorinostat (150 mg/kg) for 3 consecutive days. One day after the last injection tumors were excised and single cell suspensions were made. CD45.2+CD11b+F4/80low and CD45.2+CD11b+F4/80high cells were gated and analyzed for the expression of FcRγ1 and FcRγ2/3. Representative data from 3 independent experiments are shown.
