# Supplementary material for: Anti-GD2 mAb and Vorinostat synergize in the treatment of neuroblastoma
Source: Oncoimmunology. 2016 Mar 28;5(6):e1164919. doi: 10.1080/2162402X.2016.1164919 (PMC4938306; doi:10.1080/2162402X.2016.1164919)
Supplement: KONI_A_1164919_s02.zip [file koni-05-06-1164919-s001.zip › 2015ONCOIMM0693R-f07-z-bw.pptx]

## Slide 1
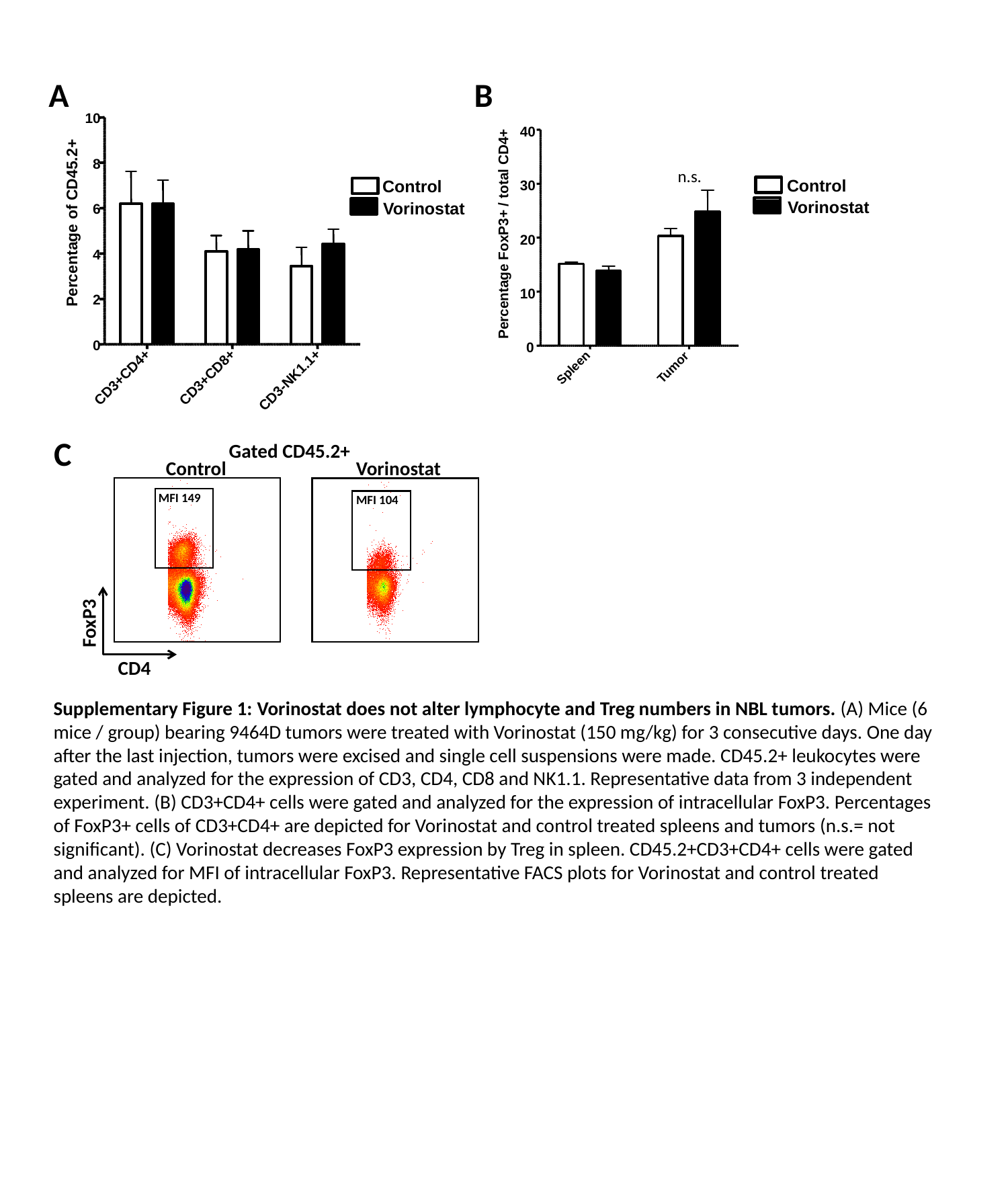

B
A
10
CD3+CD4+
CD3+CD8+
CD3-NK1.1+
8
Control
Vorinostat
6
Percentage of CD45.2+
4
2
0
40
Tumor
Spleen
30
Percentage FoxP3+ / total CD4+
20
10
0
Control
Vorinostat
n.s.
C
Control
Vorinostat
MFI 149
MFI 104
FoxP3
CD4
Gated CD45.2+
Supplementary Figure 1: Vorinostat does not alter lymphocyte and Treg numbers in NBL tumors. (A) Mice (6 mice / group) bearing 9464D tumors were treated with Vorinostat (150 mg/kg) for 3 consecutive days. One day after the last injection, tumors were excised and single cell suspensions were made. CD45.2+ leukocytes were gated and analyzed for the expression of CD3, CD4, CD8 and NK1.1. Representative data from 3 independent experiment. (B) CD3+CD4+ cells were gated and analyzed for the expression of intracellular FoxP3. Percentages of FoxP3+ cells of CD3+CD4+ are depicted for Vorinostat and control treated spleens and tumors (n.s.= not significant). (C) Vorinostat decreases FoxP3 expression by Treg in spleen. CD45.2+CD3+CD4+ cells were gated and analyzed for MFI of intracellular FoxP3. Representative FACS plots for Vorinostat and control treated spleens are depicted.
